# Memory for Self-Performed Actions in Individuals with Asperger Syndrome

**DOI:** 10.1371/journal.pone.0013370

**Published:** 2010-10-12

**Authors:** Tiziana Zalla, Elena Daprati, Anca-Maria Sav, Pauline Chaste, Daniele Nico, Marion Leboyer

**Affiliations:** 1 Institut Jean Nicod, CNRS, Ecole Normale Supérieure, Paris, France; 2 Dipartimento di Neuroscienze and Centro di Biomedicina Spaziale, Università Roma Tor Vergata, Roma, Italy; 3 Dipartimento di Fisiologia Neuromotoria, IRCCS Fondazione Santa Lucia, Roma, Italy; 4 Laboratoire EA 2027, University Paris VIII, Paris, France; 5 Psychiatrie de l'Enfant et de l'Adolescent, Hôpital Robert Debré, Paris, France; 6 Dipartimento di Psicologia, Università La Sapienza, Roma, Italy; 7 INSERM U 955, IMRB and University Paris Est Creteil, AP-HP, Henri Mondor-Albert Chenevier Hospitals, Department of Psychiatry, Fondation FondaMental, French National Science Foundation, Creteil, France; University of Regensburg, Germany

## Abstract

Memory for action is enhanced if individuals are allowed to perform the corresponding movements, compared to when they simply listen to them (enactment effect). Previous studies have shown that individuals with Autism Spectrum Disorders (ASD) have difficulties with processes involving the self, such as autobiographical memories and self performed actions. The present study aimed at assessing memory for action in Asperger Syndrome (AS). We investigated whether adults with AS would benefit from the enactment effect when recalling a list of previously performed items vs. items that were only visually and verbally experienced through three experimental tasks (Free Recall, Old/New Recognition and Source Memory). The results showed that while performance on Recognition and Source Memory tasks was preserved in individuals with AS, the enactment effect for self-performed actions was not consistently present, as revealed by the lower number of performed actions being recalled on the Free Recall test, as compared to adults with typical development. Subtle difficulties in encoding specific motor and proprioceptive signals during action execution in individuals with AS might affect retrieval of relevant personal episodic information. These disturbances might be associated to an impaired action monitoring system.

## Introduction

Autism spectrum disorders (ASD) are pervasive developmental disorders characterized by abnormal social interaction, verbal and non-verbal communication problems and restricted interests. Within the domain of ASD, High functioning autism (HFA) commonly refers to individuals meeting criteria for autism with normal intellectual functioning and a history of speech and language delay. Those at the higher-functioning end of the HFA group, sometimes diagnosed with Asperger Syndrome (AS) [1], [2] show no evidence of delayed language function and their intellectual abilities fall within the normal range. As with other individuals with ASD, the clinical features of HFA and AS include troubles forming friendships, difficulties with social cognition, inappropriate social interactions, poor communication, restricted interests and diminished capacity for empathy. Importantly and contrary to the more severe forms of autism, individuals with AS may pass tests of Theory of Mind (ToM), i.e., tests evaluating the ability to attribute mental states, such as intentions, beliefs and desires, to oneself and others [3]. Specifically, individuals with AS can often solve first-order (e.g. “Sally thinks it's x, when really it's y”) and second-order false beliefs tests (e.g. “Sally thinks Mary thinks x, but both Sally and Mary are wrong”) [4], [5], although they might fail more ‘advanced’ ToM tasks, based on detection of sarcasm, irony or bluff [6] or on recognition of Faux Pas [7], [8].

In recent years, there have been relatively few experimental studies on action memory in adults with ASD and many findings remain controversial, revealing a pattern of both spared and impaired capacities. Individuals with HFA or AS are often described as endowed with prodigious memory capacities, and capable of memorizing large quantities of information [9]. Immediate memory span [10], cued recall [11], [12] and recognition seem to be preserved, at least in autistic individuals without global cognitive impairment [13]. However, other studies have found that free recall is often impaired and moderate impairments in episodic memory have been reported in these individuals for tasks requiring a high degree of attentional control, or the use of complex organizing strategies [14], [15], [16]. More recently, adults with AS were found to recall fewer specific details in autobiographical memories, and to express their identities using significantly more abstract trait-linked statements than comparison participants [17], [18].

Previous studies also indicate that individuals with ASD may be less accurate on source monitoring tasks [19]–[22]. Source monitoring refers to the ability to recall the origins of memories, knowledge and beliefs and involves the spatiotemporal context under which a memory is acquired. It is thought to be related to the episodic memory system and to play a crucial role in discriminating self-other information [23]. However, other studies have shown that some types of source monitoring ability are preserved in individuals with ASD with an otherwise normal cognitive profile [24] or when appropriately supported testing procedures are used during recall [19].

Russell and Jarrold [25] explained difficulties with memory for self-performed actions in a group of children with autism in terms of monitoring deficits. The authors required participants to remember whether they or the experimenter had placed a picture card on a grid, either on their own behalf or on behalf of a doll partner. They argued that difficulties in recalling whether a placement had been made by themselves or another individual in children with autism would be due to the failure to monitor their actions as their own. However, Hill and Russell [26] attempted to replicate Russell and Jarrold's experiment, but reported intact self-other source attribution in children with autism. More recently, Williams and Happé [27] found that, when compared to IQ-matched comparison participants, individuals with ASD recall their own actions better than those of the experimenter showing a typical self-reference effect. In contrast with this finding, other studies have shown a reduced self-reference effect in individuals with ASD [28]–[30]. Millward and collaborators [28] reported that children with autism have a specific difficulty with the recall of personally experienced events, as compared with memory for events experienced by a peer. Using a recognition test, Toichi and collaborators [29] showed that adults with HFA do not benefit from the self-reference effect since they are impaired in processing words in a self-related manner, in the absence of semantic and phonological impairments. More recently, Hare, Mellor and Azmi [30] found that adults with ASD demonstrate superiority for self-experienced events over events merely observed when the recall is cued whilst this superiority effect disappeared in free recall. Despite these contradictory results, substantial evidence supports the notion of a diminished memory for personal knowledge, although the degree to which this impairment relates to inadequate encoding, action monitoring impairments, introspective limitations or faulty recall remains poorly understood. In fact, there are some grounds for supposing that difficulties with memory for self-performed action in individuals with ASD might relate to an executive dysfunction, and in particular to action monitoring impairments [31], [32].

The ‘Central Monitoring Theory’ is a predominant account on explaining impairments in motor learning and motor control [33], [34]. According to this theory, internal models are implemented in the central motor system. Predictive models use efferent copies that predict the sensory consequences of a given motor command, which are eventually compared to the actual sensory outcome [35]. The matching between central motor signals and visual, tactile and proprioceptive feedback that arise during action execution, together with the associated action intention, is thought to be a crucial mechanism involved in action monitoring. This mechanism might fail in ASD [36].

It is well known that motor and proprioceptive signals preponderantly function in the absence of awareness and might thus covertly affect memory strength [37]. In particular, Engelkamp [38] has demonstrated that the processes related to motor performance provide verbal memories with more durable representations than those received from external sources (i.e. action observation or verbal semantic description). Specifically, memory performance for a list of simple action sentences (e.g., open an umbrella) is increased if the listener simultaneously executes the corresponding action. The memory enhancement for enacted compared to visually or verbally encoded items - called the enactment effect – [38] is effective in terms of both accuracy and processing speed of the retrieved information. This facilitation relies on a form of procedural learning that implicitly favors the enacted items, being relatively independent of conscious access to the encoded information. In agreement with these observations, people are better at recalling events that they have personally experienced compared to events experienced by another person to whom they attended [39].

The present study aimed at assessing whether adults with AS would benefit from the enactment effect when recalling a list of previously enacted items vs. items that were only visually and verbally experienced. The presence of this memory facilitation was tested through a Free Recall test and an Old/New Recognition task. Furthermore, we investigated whether these individuals can overtly distinguish self-performed actions from actions performed by others through a Source Memory Recognition task. Adults with AS exhibited a reduced enactment effect on the Free Recall test, while on the Recognition and the Source Memory tasks their performance was comparable to that of adults with typical development. These results may be explained in terms of an impaired action monitoring system, likely associated with difficulties in encoding specific motor signals during action execution that would affect retrieval of relevant personal episodic information.

## Materials and Methods

### Ethics Statement

The present research has been approved by the local Ethical committee (Inserm, C07). All participants signed informed consent before volunteering for this study, and all investigation has been conducted according to the principles expressed in the Declaration of Helsinki.

### Participants

Eighteen adults with a clinical diagnosis of Asperger Syndrome (AS) according to DSM-IV R [1] and ASDI (Asperger Syndrome Diagnostic Interview) [40] were recruited from Albert Chenevier Hospital in Créteil (see Table 1 for details). The inclusion criteria for the sample were based on retrospective parental information about the early language development of their child. All diagnoses were made by experienced clinicians and were based on clinical observations of the participants. Interviews with parents or caregivers using the ADI-R (Autism Diagnostic Interview) [41] confirmed the diagnoses. The cut-off points for the three classes of behaviour are reciprocal social interaction 10, communication 8, and stereotyped behaviours 3, respectively. All participants scored above the cut-off points.

**Table 1:** Means (and standard deviations) of demographic and clinical data for two groups (Asperger and Comparison).

	Asperger	Comparison
**N** (male∶female ratio)	15∶3	14∶4
**Age in years** (mean, SD, range)	26.2 (7.8); 17–39	27.7 (4.9); 22–40
**Education in years (mean, SD)**	15.5 (3)	15.4 (4)
**Full scale IQ**	107.4 (21.7)	106.7 (14.6)
**Verbal IQ**	114.2 (23.2)	115.8 (14.7)
**Performance IQ**	99.5 (17.4)	105.3 (8.5)
**ADI [B,C,D]**	18.7[5.2]; 11 [5.8]; 7.1 [3.2]	
**Grober & Buschke Test**		
Immediate Recall^1^	15.8 (0.3)	
Free Recall (1st; 2nd; 3^rd^)	9.4 (2) 11.7(2.2) 13.5 (1.8)	
Cued Recall (1st; 2nd; 3rd)	5.3 (1.9) 4.1(1.8) 2.3 (1.7)	
**Faux Pas Test** 2 (total score)	40.3 (8.7)	54.3 (5.7)

1 Mean values (SD); normal scores>9 (max score = 16).

2 Max score = 60.

As part of the checking process, the French translation of A-TAC (Autism, tics, AD-HD and other comorbidities) [42] was completed by the parents. This screening questionnaire is focused on a number of abilities, conducts and behaviours in a child's functioning as compared to his or her peers. Parents are asked to report any problem or specific characteristic observed at any period of life, even when this is no longer present.

Eighteen comparison participants with typical development volunteered to match the clinical group with respect to age, IQ and gender (see Table 1 for details). Prior to their recruitment, the comparison participants were screened to exclude any with a history of psychiatric or neurological disorders. All participants were native French speakers, and had normal/corrected to normal vision.

All participants received basic neuropsychological screening, which included Verbal and Performance IQs (WAIS-III) [43]. All participants had an IQ above 70. Overall, individuals with AS did not differ from the comparison participants on chronological age (t-test: t(34) = .71, p = .48, r = .11), IQ level (Full-scale, Verbal and Performance: t-test: t(30) = −0.10, p = .91, r = −.01; t(30) = .23, p = .82, r = .04; t(34) = 1.14, p = .26, r = .19). The group with AS also underwent an exploration of verbal memory functions through the French adaptation of the Grober & Buschke Test [44] and scored within the normal range. To evaluate mindreading abilities, participants were administered an advanced ToM task, the Faux-pas Recognition Test [8]. Here, the group with AS scored significantly lower than the comparison group (t(30) = 4.98, p<.0001, r = .67), consistently with what is expected from the clinical presentation of the syndrome (see Table 1). Data from three comparison subjects were excluded from analysis because they did not conform to the selection criteria.

### Procedure

**Figure 1:**
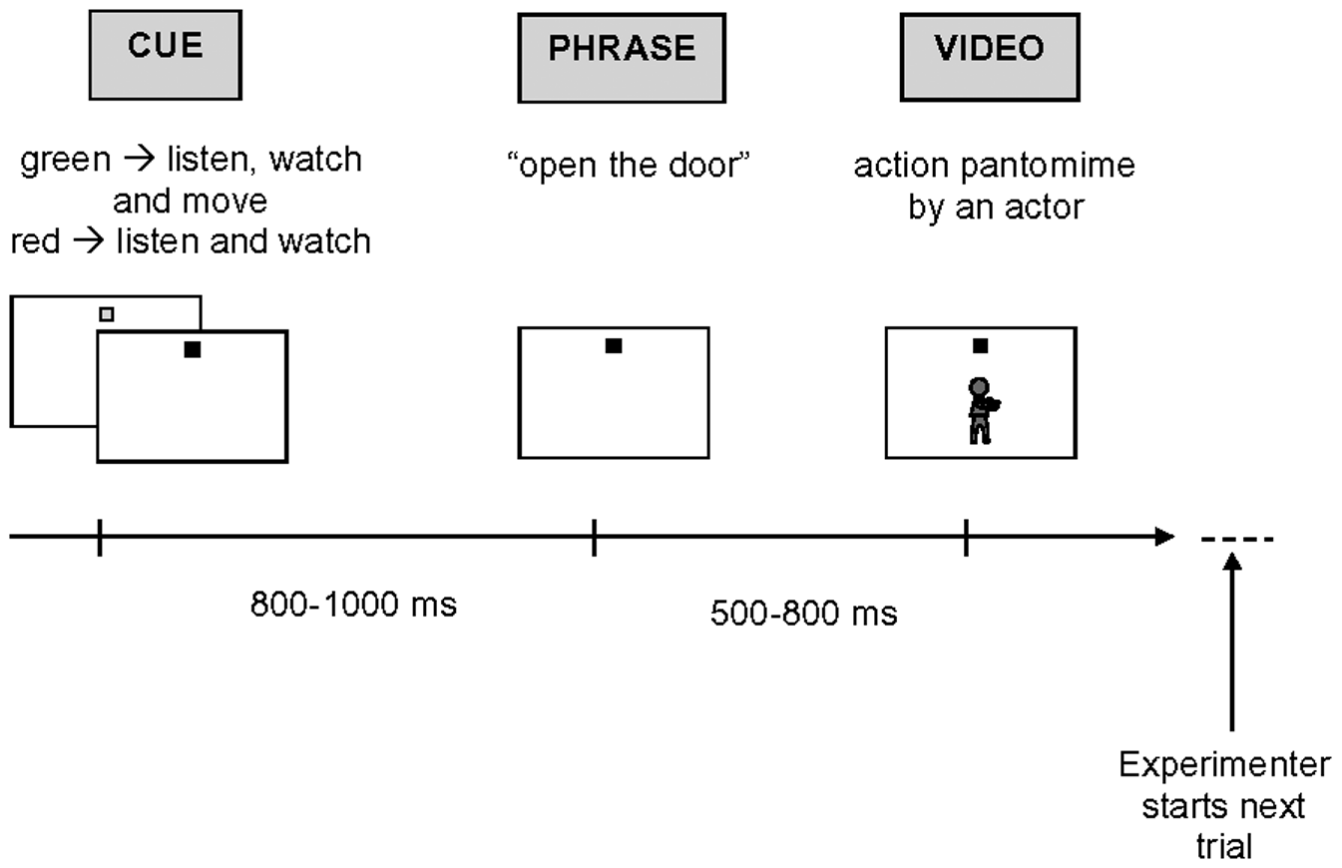
Schematic description of the procedure applied in part I.

The experiment consisted of two parts that were run in one session, with a brief interval in between. Instructions for the two parts were given separately to the participants (for details see also [45]. In part I, participants stood in front of a computer screen located approximately 1.5m from their frontal plane. They were informed that a red or a green dot (2×2cm) would appear on the upper third of the screen. This event would be followed by a recorded male voice describing an action sentence, and next by the video of an actor pantomiming the corresponding action (see Figure 1 for details).

Participants were asked to listen to the sentence and according to the dot's colour (which was meant as a cue), either watch the pantomimed action (red dot) or simultaneously execute the movement described by the sentence (green dot). They were informed that in part II, they would be interviewed about what they heard, saw and performed, but no explicit reference to a formal memory test was ever made. Eight 30-item lists were created, each including 15 to-be-enacted items (enacted) and 15 to-be-observed ones (observed). Each trial was triggered by an experimenter.

Order of list presentation was fully counterbalanced across participants. All lists were drawn from a pool of 60 action phrases and were comparable in terms of length and frequency of use of the corresponding words (see Appendix S1).

In part II, three separate measures of memory for action sentences were collected in the following sequence. First, a Free Recall test, in which participants were required to write down all the sentences they remembered from part I, by reporting the items as accurately as possible. A time limit of 5-min was given for task completion. The second measure was an Old/New Recognition task, in which participants viewed a list of 60 sentences that appeared one at a time, in the centre of a computer screen. For each sentence, participants decided whether it corresponded to an item that had previously appeared in part I (old item) or not (new item). Participants responded by pressing one of two adjacent keys (new, old) as fast and as accurately as possible. The last measure was a Source Memory task, in which participants viewed the 60-sentence list a second time and decided whether old items corresponded to enacted or observed items. Response was given by pressing one of three keys (new, enacted, observed) as fast and as accurately as possible.

Part I and II, and the three measures of recall were separated by short intervals (5 to 10 min each), during which participants were engaged in a visuo-spatial task.

### Data collection and analyses

In part I, performance of participants was monitored by two experimenters. Performance was scored as correct based on the fact that pantomime was recognizable, and did not include spatial or temporal errors or use of hands as objects, and similar mistakes. If errors occurred, the trial was singled out to be discarded. Procedural errors were absent in both the comparison and the experimental group. In part II, responses for the three memory tasks were collected and analysed as follows.

For Free Recall, items were scored as correct when they corresponded to the original sentence. Differing from the original procedure [45], minor changes in the sentence were accepted (i.e. plural instead of singular, and the like). Accuracy measures were computed as a proportion of correct answers out of the total number of presented items. Proportion of correct responses was then submitted to arcsine transformation in order to meet criteria for parametric analyses.

For the Recognition Task, data were analysed using the non-parametric indices of item discrimination A′ and response bias B″_D_ [46], computed according to equations (1) and (2) shown below:
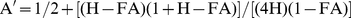
(1)

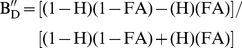
(2)where H indicates hit rates (i.e. correct choice of response ‘old’ for an old item) and FA refers to false alarms (i.e. incorrect choice of response ‘old’ for a new item). According to this procedure, an A′ value equal to 1.0 represents maximum accuracy; a value of 0.5 indicates chance-level performance. B″_D_ values less than zero represent a bias towards responding ‘old’ to all items; B″_D_ greater than zero suggests a tendency towards classifying all items as ‘new’. In both cases, the greater the B″_D_ score, the greater the bias. Separate hit rates were computed for enacted and viewed items and separate A′ and B″_D_ were computed accordingly. For false alarms, a single false alarm rate was used, similarly to what is described in a previous study using a comparable paradigm [22].

For the Source Memory Task, the number of correct source attributions was computed as a proportion of the number of hits. This was done separately for enacted and observed items.

To assess group differences, A′ and B″_D_ scores, and accuracy scores on the Free Recall and Source Memory Task were submitted to separate 2×2 ANOVAs, with group (AS, comparison participants) as between-subjects factor and type of encoding (enacted, observed) as within-subjects factor. The Scheffè test was used for post-hoc comparisons. For these statistics, the alpha level for acceptance was set at 0.05.

## Results

### Free Recall Task

**Figure 2:**
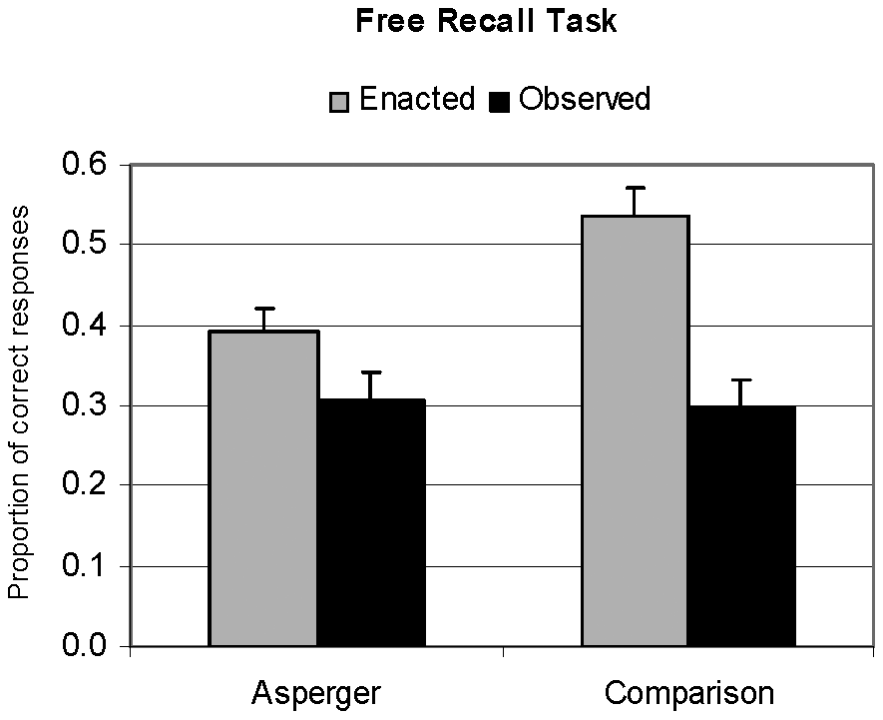
Mean proportion of correctly recalled items on the Free Recall Task for the two groups (Asperger and Comparison). The bars represent means and the whiskers represent standard errors.

On average, comparison participants freely recalled about 42% of the actions presented in part I; this percentage decreased to 35% in participants with AS. In detail (Table 2 and Figure 2), comparison participants correctly reported more enacted than observed items, while individuals with AS reported a comparable (and overall minor) number of items from both conditions. A 2-way ANOVA on proportion of correct recalls revealed no main effect of group (F(1,34) = 3.51, p = .065, r = .25), but a highly significant effect of type of Encoding (F(1,34) = 44.45, p<.0000001, r = .75), and a significant interaction between Group and type of Encoding (F(1,34) = 9.14, p<.005, r = .46). Type of Encoding affected recall, with a greater number of enacted items being recalled. The interaction was due to participants with AS recalling significantly fewer enacted items compared to comparison participants (mean diff. = .14; p<.009), whereas the proportion of the observed items was comparable in the two groups (mean diff. = −.018; p = .958) (Figure 2). In addition, a significant difference between proportions of enacted vs. observed encoded items emerged only for comparison participants (mean diff. = .24; p<.0003), whereas no difference between Encoding conditions was found for the group with AS (mean diff. = .08; p = .112).

**Table 2:** Mean values (and standard deviations) for the three memory tasks (Free recall, Recognition, Source Memory) and the two groups (Asperger and Comparison).

	Asperger		Comparison	
	Enacted	Observed	Enacted	Observed
**Free recall**	0.39 (0.13)	0.31 (0.14)	0.54 (0.14)	0.30 (0.16)
**Recognition**				
**A′ (discrimination)**	0.95 (0.04)	0.92 (0.07)	0.98 (0.02)	0.94 (0.03)
**B″D (bias)**	−0.13 (0.76)	0.42 (0.58)	−0.27 (0.71)	0.61 (0.35)
**Source memory Hit rates**	0.93 (0.08)	0.84 (0.14)	0.97 (0.04)	0.84 (0.09)

### Recognition Task

**Figure 3:**
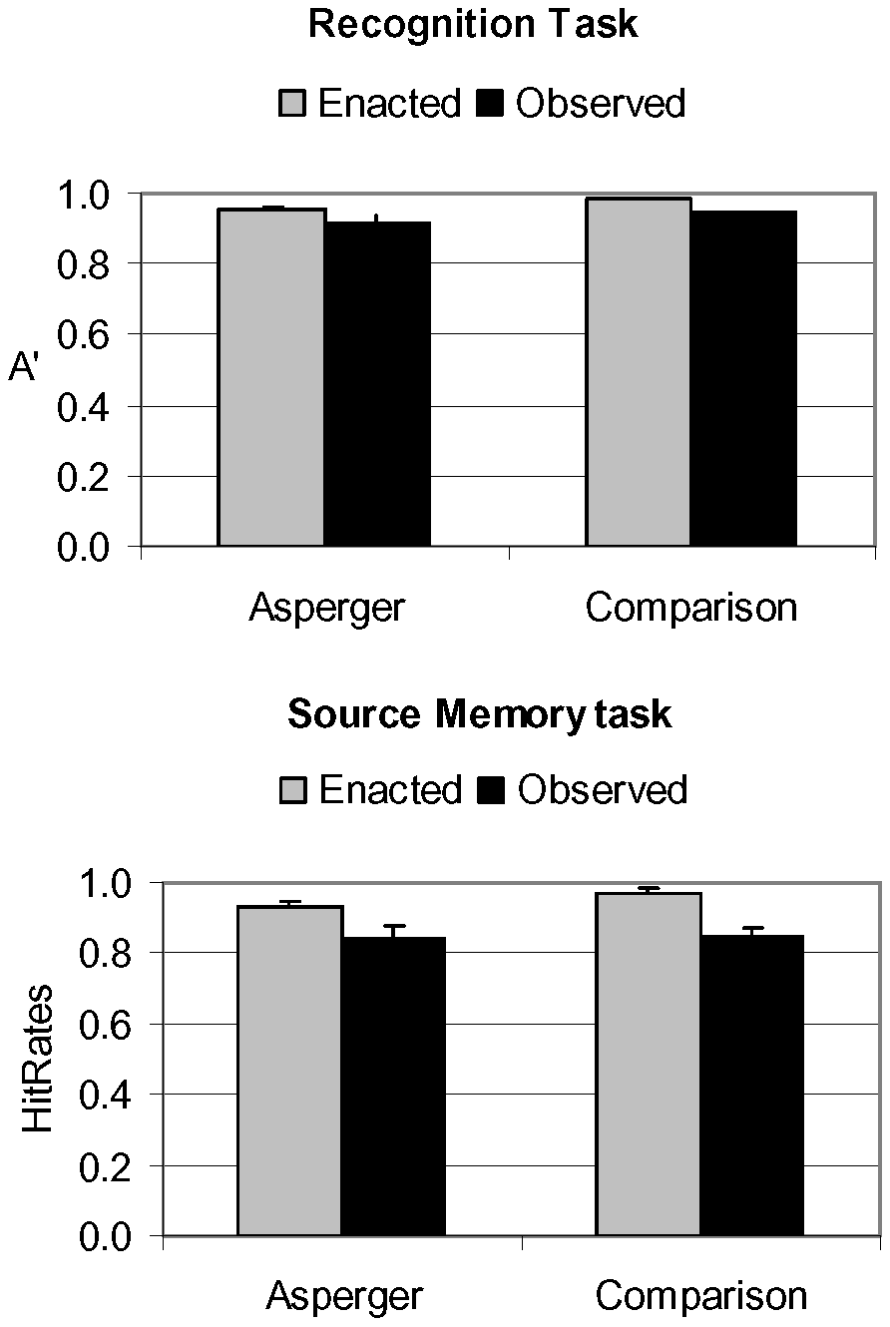
Accuracy in the Recognition and Source Memory tasks for the two groups (Asperger and Comparison). In the upper panel, A′ is given as index of discrimination abilities (the larger the index the more accurate the performance), in the lower panel, hit rate is provided. The bars represent means and the whiskers represent standard errors.

The ANOVA on A′ (discrimination) scores showed a highly significant type of Encoding (F(1,34) = 29.28, p<.000005, r = .69), but neither main effect of group (F(1,34) = 3.36, p = .075, r = .32) nor significant interaction (F(1,34) = .083, p = .77, r = .73). Both AS and comparison groups were very accurate and correctly recognized a comparable proportion of items (mean = .94; SD = .05 and mean = .96; SD = .02, respectively). Type of Encoding affected responses: observed items (mean = .93; SD = .02) led to significantly fewer correct responses compared to enacted ones (mean = .97; SD = .06) (Table 2 and Figure 3, upper panel). In view of the excellent performance of the two groups, we verified that A′ scores significantly differed from a ceiling response (i.e. 100% correct detections) by running separate t-tests on enacted and observed items. All p-values were below .001.

The ANOVA on B″_D_ (bias) scores showed only a significant main effect of type of Encoding (F(1,34) = 26.36, p<.00001, r = .67), whereas the main effect of group (F(1,34) = .0006, p = .97, r = .32) and the interaction between Group and type of Encoding (F(1,34) = 1.31, p = .26, r = .77) were not significant. The two groups showed a similar bias, which was stronger for observed items compared to enacted ones (observed: mean = .52; SD = .47; enacted: mean = .20; SD = .73). Separate t-tests were run to assess whether B″_D_ scores were significantly above zero (a B″_D_ score of zero representing an absence of bias). Results confirmed a genuine positive bias for observed items only (comparison participants: t(20) = 8.063, p<.0000001, r = .87; participants with AS: t(17) = 3.026, p<.008, r = .59), i.e. a conservative bias towards responding ‘new’ to the presented sentences.

### Source Memory Task

The ANOVA on accuracy scores yielded no group difference (F(1,29) = .95, p = .34, r = .18), but a significant main effect of type of Encoding (F(1,29) = 27.50, p = .000001, r = .70) (Table 2 and Figure 3, lower panel). Overall, enacted items (mean = .95; SD = 06) led to a greater proportion of correct responses, as compared to observed items (mean = .84; SD = .12). No significant interaction was observed.

An ANOVA comparing overall hit rates for Recognition and Source Memory tasks in participants with AS shows a main effect of task (F(1,13) = 13.32; <.0029, r = .71). Hit rate was superior on the first task (Recognition: M = .84; SD = .12), as compared to the second task presented (Source Memory: M = .74; SD = .19).

### Correlation analyses

Correlations between participant's recall score, IQ level, and score on the Grober & Buschke Test were computed to determine whether performances on Free Recall and Recognition tasks were related to Verbal memory and Verbal IQ as well as to Performance IQ. Bonferroni corrected p was set at .003. No significant correlations emerged. Moreover, six separate correlation analyses (Pearson Product Moment test) were performed between memory measures for enacted and observed items on the three experimental tasks (Free Recall, Recognition and Source Memory) and ToM ability, as assessed by the Faux Pas task. Bonferroni corrected p was set at .008. No significant correlations emerged.

## Discussion

The present results showed the absence of enactment effect, i.e. a positive difference between the proportions of enacted vs. observed (visually and verbally encoded) items in individuals with AS. Interestingly, this result emerged only when they were engaged in voluntary retrieval of previously presented items (i.e. Free Recall task), suggesting that participants with AS did not benefit from performing the actions to the same extent as participants with typical development. In fact, when successively tested on New-Old and Source Memory Recognition tasks, the two groups showed similar performance. We believe that these findings are poorly explained in terms of a general episodic memory deficit and that the absence of an enactment effect on the Free Recall task in individuals with AS might reflect two possible impairments: either they have lost the ability to store the distinctive cues that characterize self-generated events or they might fail to access self-relevant information. We will discuss these issues in detail.

### A diminished enactment effect: loss of privileged ‘self’ status?

Although memory impairments are reported in autism and Asperger Syndrome [11], [14], [15], [18], [24], it is unlikely that the present findings can be explained as stemming from a general episodic memory deficit. Indeed, recall of other-performed (visually and verbally encoded) actions was unaffected in our group with AS and, although only one measure was investigated here (i.e. the Grober and Buschke test), verbal memory was within the normal range. In addition, no correlation emerged between measures on Recall and Recognition tasks and Verbal/Performance IQ scores or Verbal Memory abilities. Similarly, the improved performance of individuals with AS on the Recognition and Source Memory tasks, as compared to Free Recall, is unlikely to depend on the repeated presentation of the items. Were this the case, one would expect a learning effect, namely that the blocked order of task presentation would induce a progressive increase in response accuracy, reinforcing correct old/new recognition and attenuating source memory impairments. This was not found: direct comparison of the proportion of hit rates on the Recognition and Source Memory tasks rather suggests the opposite pattern, namely a better performance on the first rather than on the second task. Hence, the improved performance on the Recognition and Source Memory tasks, which require the retrieval of contextual elements from episodic memory to a lesser extent, might be due to the AS group's employing explicit recollection strategies based on a preserved semantic memory. Accordingly, the lack of enactment effect they exhibited on the Free Recall task would reflect specific difficulties in constructing personal episodic traces. This might be due to a failure to properly encode motor and proprioceptive information which would selectively impair certain aspects of personal episodic memory, along with a preserved verbal semantic memory [15], [16], [22].

Why would individuals with AS benefit less from the distinctive cues characterizing self-generated events? While memory traces for self-performed actions are more salient because they involve an additional motoric component, individuals with AS might not entirely benefit from information associated with action execution, such as efferent or central motor signals or reafferent feedback signals from proprioception. The matching between central signals, motor reafferences, and visual and proprioceptive feedbacks, is a crucial mechanism involved in action monitoring [34]. The argument is that because individuals with AS fail to integrate these signals, they do not monitor their actions as their own and do not benefit of memory enhancement for self-performed actions. It is also possible that a defective sensori-motor integration might reflect a disrupted “binding mechanism”, i.e. the process responsible for associating visual and sensori-motor information related to the self to semantic information pertaining to the action [47]. In this view, episodic information could be only partially integrated and, consequently, visual and semantic information might overwhelm sensorimotor signals, leading to a substantial similarity between enacted and observed actions.

Similarly, Russell and Jarrold [32] have suggested that insufficient monitoring of self-performed actions in autism would be associated with an impaired ability to relate motor commands to their visual outcomes by means of visual action schemata. Difficulties in anticipating the sensory consequences of one's motor output [48], as well as in motor planning [31], [49] and action prediction [50] have already been reported in individuals with ASD. In particular, using electromyographic (EMG) recordings, Cattaneo and collaborators [48], have shown that, unlike children with typical development, no EMG activity of the mouth muscle was found in children with ASD during the execution of first phase of the action sequence, and a delayed activation only appeared during the last phase, suggesting that they were unable to anticipate their own action.

Nevertheless, the existing evidence in favour of an action monitoring deficit is somewhat inconclusive [22], [27], [31], [32]. In a previous study, Lind and Bowler [22] have shown an impaired self-other source memory along with an undiminished recognition memory and a preserved enactment effect in children with ASD. These discrepancies might be explained by differences between the two samples (chronological age and IQ scores) as well as in task design. While Lind and Bowler's task relied on the ability to recall who picked up and named a given picture card (i.e. the participant or the experimenter), in our experiment the enactment effect relies on the memory traces for self-performed actions which have a different salience in terms of the specific motor component involved. Similarly, the source memory task used in Lind and Bowler's study taps on the ability to encode particular items (e.g., the card picture and the picture name) in self-relevant ways, while in our study the source memory task implies the ability to distinguish self-performed from other-performed actions, each characterized by distinctive motor components.

Difficulties in self-others source memory and action monitoring have not consistently been found when on-line discrimination of one's own actions from those of an external agent was required [26], [27]. In particular, Williams and Happé [27] reported intact action monitoring, as well as a typical self-reference effect in recalling their own actions in participants with ASD. However, according to the authors, the employment of relatively more able individuals, together with overt verbal commentaries, strongly encouraged by the experimenter, might account for the self-reference effect displayed by participants with ASD, and thus for the discrepancies with previous literature [28]–[30].

### A diminished enactment effect: failure to access self-relevant information?

On a different view, the lack of enactment effect on the Free Recall task, reported here in individuals with AS, might reflect an inability to access self-relevant information, a disturbance that has been related to a ToM deficit, i.e., the ability to represent others' and one's own intentions, beliefs and experiences [51], [52]. However, the present findings could be hardly explained by a deficit in ToM since, if this was the case, one would expect to find a more severe difficulty with tasks in which self-other attribution is explicitly required, as on the Source Memory task. In addition, no correlation emerged between measures of recall and source memory and scores on the Faux Pas test, an advanced ToM task. Indeed, autobiographical reports suggest that individuals with AS do have access to their own memories and past experiences, although self-awareness and introspective reflection are phenomenologically different from those observed in people with typical development. Episodic autobiographical memories in individuals with ASD often have a “perspective-free” character; they may be fewer in number and lacking in specific details [17], [18], or predominantly (and even exclusively) visual in content [53]. These findings are in accordance with the view that subtle impairments in encoding distinctive motor and proprioceptive cues during action execution might selectively affect personal episodic memory. Thus, individuals with AS do not show an implicit facilitation effect for self-performed actions on Free Recall, and only show a retrieval advantage for these events when recall is cued [c.f.], [ 30,54], or on recognition tasks in which more explicit cognitive strategies based on their intact verbal abilities or semantic memory are employed [c.f.], [ 6, 22]. This view is consistent with a diminished (rather than entirely disrupted) form of autonoetic awareness (i.e. the state of remembering) along with an unimpaired noetic awareness (i.e. the state of knowing) [55].

### Concluding remarks

In conclusion, we believe that the reduced enactment effect in adults with AS reveals an impaired action monitoring system, likely associated with an insufficient use of motor and proprioceptive information or with an inadequate sensorimotor integration. Subtle difficulties in encoding specific motor signals during action execution might affect retrieval of relevant personal episodic information, as well as the development of an extended sense of self in individuals with AS. Future studies are needed to further investigate this hypothesis by carefully and systematically varying visual, motor and efferent signals during action execution tasks.

## Supporting Information

Appendix S1 (0.02 MB DOC)
