# Supplementary material for: Memory for Self-Performed Actions in Individuals with Asperger Syndrome
Source: PLoS One. 2010 Oct 12;5(10):e13370. doi: 10.1371/journal.pone.0013370 (PMC2953510; doi:10.1371/journal.pone.0013370)
Supplement: Appendix S1 — (0.02 MB DOC) [file pone.0013370.s001.doc]

### Appendix S1

*Complete phrase list of action phrases used in the experiment.*

Jouer aux casino (to play slot-machines); peindre un mur (to paint a wall), jouer au basket (to play basket-ball), tourner une clef (to turn a key), repasser un drap (to iron a cloth), porter une valise (to carry a suitcase), ouvrir une porte (to open a door), tourner des pages (to turn pages), jouer aux billes (to play marbles), verser de l’eau (to pour water), caresser un chien (to pat a dog), jouer aux dés (to play dice). secouer un shaker (to shake a shaker), ouvrir un store (to open a blind), fermer un bocal (to close a jar), déchirer une feuille (to tear a sheet of paper), jouer de la batterie (to play drums), étaler la pâte (to spread dough), couper de la viande (to cut meat), visser une ampoule (to screw in a light bulb), distribuer les cartes (to deal cards), planter un clou (to fix a nail), coudre un bouton (to sew on a button), scier du bois (to saw wood). se peigner les cheveux (to comb one’s hair), répondre au téléphone (to answer the phone), mettre un chapeau (to put one’s hat on), s’essuyer la bouche (to wipe one’s mouth), mettre des lunettes (to put on glasses), boire à la bouteille (to drink from the bottle), manger de la soupe (to eat soup with a spoon), boutonner sa chemise (to button one’s shirt), mettre une écharpe (to put on one’s scarf), se couper les ongles (to cut one’s nails), enfiler un gant (to put on a glove), mettre sa montre (to put on one’s watch). Tourner la tête (to turn one’s head), regarder en l’air (to look up), souffler très fort (to blow very hard), se gratter le nez (to rub one’s nose), se boucher les oreilles (to cover one’s ears), se recoiffer le cheveux (to do/fix one’s hair), étirer les bras (to stretch one’s arms), se pencher en avant (to bend over), tourner sur soi (to rotate), se frotter le bras (to rub one’s arm), se frotter les mains (to rub one’s hands), se craquer les doigts (to crack one’s fingers/knuckles). hausser les epaules (to shrug), faire une reverence (to bow), se prendre la tête (to take one’s head in one’s hands), faire le salut militaire (to give a salute), faire signe de venir (to beckon someone), serrer la main (to shake hands), faire du stop (to hitch-hike), faire c’est super (to make an OK sign), claquer des doigts (to snap one’s fingers), croiser les doigts (to cross one’s fingers), compter sur ses doigts (to count on one’s fingers), se tourner les pouces (to twiddle one’s thumbs).
